# P-1115. COT-832, a Novel Polyene Macrolide Antifungal (II): *In Vivo* Fungicidal Efficacy against *Aspergillus fumigatus* and *Candida albicans* in Mice and Rats Infection Models

**DOI:** 10.1093/ofid/ofae631.1302

**Published:** 2025-01-29

**Authors:** Yuri Nishiyama, Hideki Maki, Osamu Yoshida, Yasushi Hasegawa, Yuuta Ukai, Tamio Fukushima

**Affiliations:** Shionogi & Co., Ltd., Toyonaka, Osaka, Japan; Shionogi & Co., Ltd., Toyonaka, Osaka, Japan; Shionogi & Co., Ltd., Toyonaka, Osaka, Japan; Shionogi & Co., Ltd., Toyonaka, Osaka, Japan; Shionogi & Co., Ltd., Toyonaka, Osaka, Japan; Shionogi & Co., Ltd., Toyonaka, Osaka, Japan

## Abstract

**Background:**

A novel antifungal compound, COT-832 was created by SHIONOGI through modifying Amphotericin B (AmB). Marketed AmB formulations are often discontinued in clinical use due to nephrotoxicity. In this study, we investigated in vivo efficacy of COT-832 against *Aspergillus fumigatus* (*A. fumigatus*) and *Candida albicans* (*C. albicans*) in mice and rats infection models in comparison with those of Fungizone (FGZ) and AmBisome (L-AMB) at each human clinically equivalent dose (CED) in AUC.

**Methods:**

CED;

Mice (male ICR mice): FGZ;2.2 mg/kg, L-AMB; 7 mg/kg,

Rats (Crl:CD(SD)): L-AMB; 8 mg/kg,

cf. COT-832; 4 mg/kg showed the safety level similar to L-AMB at CED in a rat 2-week toxicology study.

Systemic candidiasis in mice and rats: Conidia of *C. albicans* were intravenously administered (iv) in immunosuppressed mice. At 2, 24, and 48 hours (h) in mice, 48h in rats post-infection, each compound was administered (iv). Fungal burden in kidney was quantified at 24h post-treatment.

Invasive pulmonary aspergillosis in rats: 10^4^ conidia of *A. fumigatus* were inoculated intranasally in immunosuppressed rats. At 24h post-infection, each compound was administered (iv). *A. fumigatus* 18S rRNA in lung was quantified at 24h post-treatment.

**Results:**

Systemic candidiasis in mice: FGZ at CED; immediate death when treatment was initiated at 24 and 48h post-infection. L-AMB at CED; fungistatic when initiated at 24h post-infection. COT-832; fungicidal at 3 mg/kg when initiated at 24h post-infection.

Systemic candidiasis in rats: COT-832 at 2 mg/kg showed fungistatic efficacy comparable to L-AMB at CED, and COT-832 at 4 mg/kg showed 1-log down fungicidal efficacy from initial.

Invasive pulmonary aspergillosis in rats: COT-832 at 2 mg/kg showed comparable efficacy to L-AMB at CED, and COT-832 at 4 mg/kg showed further 1-log down fungicidal efficacy.

**Conclusion:**

In our rodent infection models, COT-832 showed more potent fungicidal effects in systemic candidiasis and invasive pulmonary aspergillosis at a dose with similar safety level to that of L-AMB at a clinically equivalent dose. These results suggest that COT-832 can be a novel antifungal active pharmaceutical ingredient to exhibit potent in vivo efficacy with less safety concerns than L-AMB.

**Disclosures:**

**Yuri Nishiyama, MS**, Shionogi & Co., Ltd.: employee **Hideki Maki, PhD**, Shionogi & Co., Ltd.: employee|Shionogi & Co., Ltd.: employee **Osamu Yoshida, n/a**, Shionogi & Co., Ltd.: employee **Yasushi Hasegawa, n/a**, Shionogi & Co., Ltd.: employee **Yuuta Ukai, MSc**, Shionogi & Co., Ltd.: employee **Tamio Fukushima, n/a**, Shionogi & Co., Ltd.: employee

